# Plasma Enhanced Complete Oxidation of Ultrathin Epitaxial Praseodymia Films on Si(111)

**DOI:** 10.3390/ma8095312

**Published:** 2015-09-18

**Authors:** Olga Kuschel, Florian Dieck, Henrik Wilkens, Sebastian Gevers, Jari Rodewald, Christian Otte, Marvin Hartwig Zoellner, Gang Niu, Thomas Schroeder, Joachim Wollschläger

**Affiliations:** 1 Fachbereich Physik, Osnabrück University, Barbarastr. 7, Osnabrück D-49069, Germany; E-Mails: oschuckm@uos.de (O.K.); fdieck@uos.de (F.D.); hwilkens@uos.de (H.W.); segevers@uos.de (S.G.); jarodewa@uos.de (J.R.); chotte@uos.de (C.O.); 2 Center of Physics and Chemistry of New Materials, Osnabrück University, Barbarastr. 7, Osnabrück D-49069, Germany; 3 Innovation for High Performance Microelectronics (IHP), Im Technologiepark 25, Frankfurt (Oder) D-15236, Germany; E-Mails: zoellner@ihp-microelectronics.com (M.H.Z.); gang@ihp-microelectronics.com (G.N.); schroeder@ihp-microelectronics.com (T.S.)

**Keywords:** praseodymia, ultrathin film, molecular beam epitaxy, plasma enhanced oxidation, strain, phase separation, X-ray photoelectron spectroscopy, low energy electron diffraction, synchrotron radiation X-ray reflectometry, synchrotron radiation X-ray diffraction

## Abstract

Praseodymia films have been exposed to oxygen plasma at room temperature after deposition on Si(111) via molecular beam epitaxy. Different parameters as film thickness, exposure time and flux during plasma treatment have been varied to study their influence on the oxygen plasma oxidation process. The surface near regions have been investigated by means of X-ray photoelectron spectroscopy showing that the plasma treatment transforms the stoichiometry of the films from Pr${}_{2}$O${}_{3}$ to PrO${}_{2}$. Closer inspection of the bulk properties of the films by means of synchrotron radiation based X-ray reflectometry and diffraction confirms this transformation if the films are thicker than some critical thickness of 6 nm. The layer distance of these films is extremely small verifying the completeness of the plasma oxidation process. Thinner films, however, cannot be transformed completely. For all films, less oxidized very thin interlayers are detected by these experimental techniques.

## 1. Introduction

Rare earth oxides (REOs) are of interest in different fields as heterogeneous catalysis [1] as well as microelectronics [2]. Thus, REO films may be used in Si technology to improve the performance and functionality of electronic devices by integrating alternative semiconductor materials (engineered wafers) [3]. For instance, germanium-on-insulator (GOI) systems are aimed to improve the speed of complementary metal-oxide-semiconductor technologies and further to achieve the cost-effective monolithic integration of III-V optoelectronic materials (GaAs) on the Si wafer platform [4].

Here, praseodymia is a promising candidate for these insulating buffer layers as demonstrated in previous studies on the growth of Ge films on praseodymia-Si(111) heterostructures [4,5]. For this purpose, it may be advantageous to use PrO${}_{2}$ films since they match very well the lattice of the Si(111) support. Both the fluorite lattice of PrO${}_{2}$ and the diamond lattice of Si are based on the fcc lattice. Furthermore, the lattice mismatch of −0.86% at room temperature is very small.

However, PrO${}_{2}$ is not stable under condition of Ultra High Vacuum (UHV) where epitaxial growth via Molecular Beam Epitaxy (MBE) has to be performed. Under these conditions, praseodymia films are deposited with Pr${}_{2}$O${}_{3}$ stoichiometry. Initially these films show hexagonal bulk structure which relaxes to the cubic structure beyond some critical film thickness [6].

Therefore, Pr${}_{2}$O${}_{3}$ films have to be exposed to O${}_{2}$ atmosphere to oxidize them and to transfer their structures to PrO${}_{2}$. Previously, Weisemoeller *et al.* [7,8] as well as Gevers *et al.* [9] demonstrated that this transformation is possible if the samples are exposed to 1 bar O${}_{2}$ at temperatures beyond 300 ${}^{\circ }$C. Closer inspection of these praseodymia films by Synchrotron Radiation based X-Ray Diffraction (SR-XRD) revealed that the films were not completely oxidized. Remaining parts showed the pseudo-cubic structure of Pr${}_{6}$O${}_{11}$.

Recently, it has been demonstrated that higher degrees of oxidation of praseodymia films can be obtained by exposure to a cold O${}_{2}$ plasma [10,11]. Studying the stoichiometry of the surface near region by X-ray Photoelectron Spectroscopy (XPS) showed that PrO${}_{2}$ stoichiometry can be obtained by this treatment [10]. Furthermore, the SR-XRD investigations showed that the lattice constants of these PrO${}_{2}$ films were smaller than ever reported before for bulk material [11].

Here, we present a detailed study on the plasma oxidation process varying the thickness of the praseodymia films as well as the time of exposure and the O${}_{2}$ flux during plasma treatment. We characterize the surface near stoichiometry by means of XPS while we use Synchrotron Radiation based X-Ray Reflectometry (SR-XRR) as well as SR-XRD to study bulk properties of the ultrathin films.

## 2. Experimental Section

After Boron doped Si(111) substrates had been cleaned with standard techniques praseodymia films have been deposited at 625 ${}^{\circ }$C by means of MBE using an e-beam evaporator and controlling the growth conditions by means of Reflection High Energy Electron Diffraction (RHEED) [6]. The Pr${}_{2}$O${}_{3}$ stoichiometry of these films has been confirmed *in-situ* by XPS. These films have hexagonal structure after deposition [12] but transform to cubic structure after Post Deposition Annealing (PDA) in 1 bar N${}_{2}$ [13] or in 10${}^{-5}$ mbar O${}_{2}$ [12].

Thereafter the samples have been transferred to a plasma chamber through air. The plasma chamber is connected to an UHV analysis chamber to characterize the surface properties of the films by means of XPS and Low Energy Electron Diffraction (LEED). We used Al K${}_{\alpha }$ emission at 1486.6 eV for the XPS experiments performed here.

During transfer through air the Pr${}_{2}$O${}_{3}$ films oxidize under ambient conditions and, thereafter, exhibit Pr${}_{6}$O${}_{11}$ stoichiometry with pseudo-cubic structure as proved by SR-XRD (see below). The praseodymia films have been annealed at 300 ${}^{\circ }$C for 2 h in UHV to remove contaminants as well as to reduce the praseodymia films and to obtain Pr${}_{2}$O${}_{3}$ stoichiometry for the films as proved by XPS (see below). LEED experiments show that the films also have pseudo-cubic structure where O vacancies are randomly distributed. The bixbyite structure with ordered O vacancies and doubled bulk unit cell could not be observed.

In addition, the plasma chamber is equipped with a microwave source working at 2.45 GHz to produce a cold oxygen plasma. The plasma oxidation of the praseodymia films has been done at room temperature. Each oxidation step has been characterized *in-situ* by means of XPS and LEED. For the oxidation process we varied the parameters of exposition time, oxygen flux and film thickness. The influence of these parameters will be discussed below.

Further *ex-situ* experiments by SR-XRR and SR-XRD have been performed at the Deutsches Elektronensynchrotron (DESY) using the beamlines W1 and P08 of the DORIS III storage ring and the PETRA III storage ring, respectively. Photons of 10.0 keV (wavelength 124 pm) and 12.4 keV (wavelength 100 pm) were used at the beamlines W1 and P08, respectively. Both beamlines are equipped with six-circle diffractometers.

## 3. Results and Discussion

The surface of the oxygen plasma treated films has been studied *in-situ* directly after treatment by means of LEED and XPS. These results are reported in the following section. Further studies on the “bulk” properties of the praseodymia films have been done by means of *ex-situ* investigations using SR-XRR and SR-XRD techniques. The results of these studies are presented in the further sections.

### 3.1. Surface Characterization

As reported above, the surfaces were characterized by XPS and LEED after initial preparation by annealing at 300 ${}^{\circ }$C. The surface diffraction pattern always showed the hexagonal pattern expected for (111) surfaces of films with pseudo-cubic bulk structure. In addition, the pattern exhibited the threefold rotational symmetry of the intensities of the higher order diffraction peaks expected due to the ABC stacking of the bulk material.

**Figure 1:**
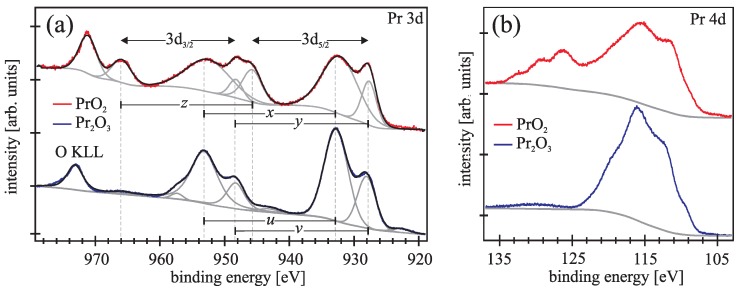
Pr 3d (**a**) and Pr 4d (**b**) photoemission spectra from praseodymia films before (blue lines) and after plasma treatment (red lines). (**a**) Bonding and anti-bonding states of Pr${}^{3+}$ (Pr${}_{2}$O${}_{3}$ stoichiometry) are denoted by *u* and *v*, respectively, while these states are denoted by *x* and *y* for Pr${}^{4+}$ (PrO${}_{2}$ stoichiometry). In addition, *z* denotes the pure main emission here; (**b**) Pr 4d photoelectrons due to higher binding energies are observed after the transformation from Pr${}_{2}$O${}_{3}$ to PrO${}_{2}$.

Typical Pr 3d and Pr 4d photoemission spectra (blue lines) obtained after the initial annealing preparation are presented in Figure 1a,b, respectively. The spectra agree well with former photoemission spectra of Pr${}_{2}$O${}_{3}$ which were calculated and analyzed in detail by Kotani and Ogasawara [14,15]. All cations in Pr${}_{2}$O${}_{3}$ have Pr${}^{3+}$ valence state. Thus, the ground state exhibits a 4f${}^{2}$ configuration, which leads in the final state of Pr3d photoemission spectra to two final states in each 3d${}_{3/2}$ and 3d${}_{5/2}$ component. The emission peaks in Figure 1a denoted by *u* and *v* are attributed to the bonding 4f${}^{2}$ and anti-bonding 4f${}^{3}\underline{v}$ configuration, respectively, which are strongly affected by the covalency hybridization induced by the core hole potential. Here $\underline{\text{v}}$ denotes a hole in the O2p valence band. The spin orbit splitting is 20.6 eV. Beyond 970 eV the Auger O KLL emission can be seen for the investigated emission range.

The Pr 4d spectrum of the Pr${}_{2}$O${}_{3}$ films presented in Figure 1a is governed by multiplet coupling effects, due to exchange interaction between 4d and 4f states. The spin-orbit-coupling in the 4d states has a minor impact than the exchange interaction between 4d and 4f states, so the signal is not separated into 4d${}_{3/2}$ and 4d${}_{5/2}$ [15].

As demonstrated by Figure 1a,b, the Pr 3d and Pr 4d photoelectron emission spectra (red lines) change drastically if the praseodymia films are oxidized by plasma treatment. Here, the ground state consists of a mixing between 4f${}^{1}$ and 4f${}^{2}\underline{\text{v}}$ configurations which leads in the final state of Pr 3d photoemission spectra to three final states in each Pr 3d${}_{3/2}$ and Pr 3d${}_{5/2}$ emission line. The peaks denoted by *x* and *y* correspond to the the bonding and antibonding final states of the strong mixed 4f${}^{2}\underline{\text{v}}$ and 4f${}^{3}\underline{\text{v}{}^{2}}$ configuration while the peaks denoted by *z* are attributed to the pure 4f${}^{1}$ main line [16]. The spin-orbit splitting is 20.6 eV, too.

Comparing the spectra obtained from the Pr${}_{2}$O${}_{3}$ and PrO${}_{2}$ films obtained before and after plasma treatment, respectively, one can clearly see differences in the spectral shape of the Pr 3d photoemission signal due to the oxidation in oxygen plasma (Figure 1a). This is a clear evidence that the surface near region of the plasma treated films are completely oxidized and exhibit the PrO${}_{2}$ stoichiometry.

The Pr 4d spectrum of the plasma treated films presented in Figure 1b range from 106 eV to 135 eV binding energy. Compared to the Pr 4d spectrum obtained from Pr${}_{2}$O${}_{3}$, there is a shift of the spectral shape from lower to higher binding energies for plasma oxidized films.

We like to emphasize that we observe very similar photoemission spectra for all plasma treatments used in our studies and for all investigated thicknesses of the praseodymia film. All spectra agree well with the spectra reported for tetravalent Pr 3d spectra in literature [10,17]. Hence, oxygen plasma treatment leads to the formation of PrO${}_{2}$ in surface near regions.

### 3.2. Film Thickness

Having performed the initial cleaning procedure, Pr${}_{2}$O${}_{3}$ films of different thickness have been exposed to oxygen plasma at 26 Pa O${}_{2}$ for 15 min using an O${}_{2}$ flux of 40 sccm.

**Figure 2:**
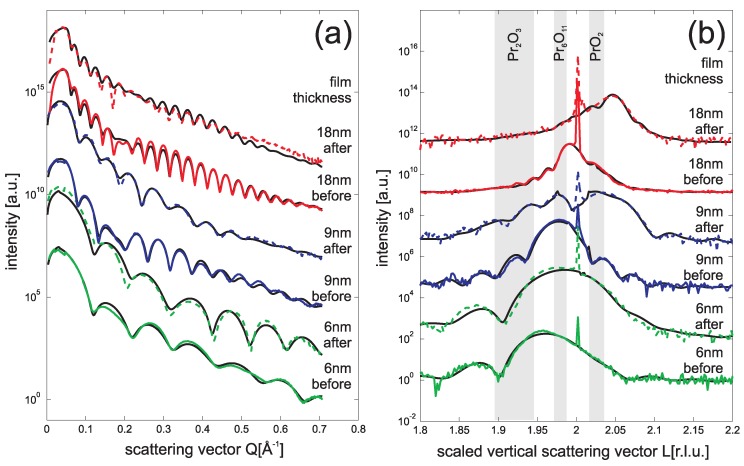
SR-XRR (**a**) and SR-XRD ((00L) crystal truncation rod) (**b**) data obtained from praseodymia films of different thickness: 6 nm (green line), 9 nm (blue line) and 18 nm (red line). The lower curves are obtained from the PDA treated samples while the upper curves are obtained after additional oxygen plasma treatment (26 Pa O${}_{2}$, 40 sccm flux, 15 min). The reference films without plasma treatment show higher oxidation states due to oxidation under ambient conditions. After plasma treatment the 9 nm and 18 nm films are complete oxidized while the 6 nm film is only partly oxidized.

Figure 2a compares SR-XRR data recorded before and after this treatment for Pr${}_{2}$O${}_{3}$ films with thickness ranging from 6 nm to 18 nm. All reflectivity curves show Kiessig fringes which have been used to determine the film thickness *a posteriori* with an accuracy better than 0.3 nm. After plasma treatment, the strength of the intensity oscillations obviously decreases for the 9 nm and the 18 nm sample while the oscillation is clearer for the 6 nm sample. This can be attributed to changes of surface roughness caused by the plasma oxidation process. The roughness of the thicker films has increased while the 6 nm film becomes smoother.

For a complete analysis of the SR-XRR data, we used the Parratt algorithm [18] combined with the model of Nevot and Croce to implement the surface roughness [19]. The fitting procedure revealed that the rms-roughness of the films is roughly 1 Å before plasma treatment. Changes of the rms-roughness after plasma treatment is very small (in the range of few 0.1 Å).

In addition, the index of refraction of the film can be obtained from the fitting procedure with an accuracy better than 2%. This result is related to the electron density of the studied praseodymia films. In the following, we assume the pseudo cubic lattice constant for the different praseodymia phases and consider their stoichiometry to calculate their electron densities. Before oxidation the praseodymia films showed an electron density of 1.80 Å${}^{-3}$. This result is very close to the calculated value of 1.78 Å${}^{-3}$ expected for Pr${}_{6}$O${}_{11}$ phase which is stable under ambient conditions. Therefore, we conclude that the original Pr${}_{2}$O${}_{3}$ films had transformed to Pr${}_{6}$O${}_{11}$ before they were inserted in the plasma chamber. However, it has to be emphasized that the films revealed Pr${}_{2}$O${}_{3}$ stoichiometry after initial annealing procedure performed in the preparation chamber before plasma oxidation (*cf.* above).

After oxidation the electron density of the praseodymia films increased to 1.90 Å${}^{-3}$. If compared to the calculated value of 1.92 Å${}^{-3}$ expected for PrO${}_{2}$, this results demonstrates the effectiveness of the plasma oxidation procedure. Within the experimental error the films are completely oxidized. This result agrees well with our XPS studies presented above. In addition, one has to conclude that PrO${}_{2}$ films are much more stable under ambient conditions than Pr${}_{2}$O${}_{3}$ films are.

Finally, we like to point to the beating effect observed in the SR-XRR data. This effect has to be attributed to the existence of an additional very thin interlayer between praseodymia film and Si(111) substrate. Our analysis shows that the thickness of this interlayers is 1.4 (±0.4) nm and that the electron density of the interlayers is 1.2 (±0.1) Å${}^{-3}$. Therefore, we conclude that the interlayers are formed by silicate due to interface reaction before. This effect is well known in literature where it is also reported that these films are amorphous [8,20].

These praseodymia films were investigated by SR-XRD, too. The results are presented in Figure 2b after scanning the (00L) Crystal Truncation Rod (CTR) close to the second Bragg condition *L* = 2. Here, we use the surface notation to describe the diffraction geometry. Thus, the vertical component $Q_{⊥}$ of the scattering vector is related to the distance $c_{\mathrm{Si}}$ of Si(111) bulk layers and *L* denotes the scaled vertical component *L* = 2$\pi Q_{⊥}/c_{\mathrm{Si}}$.

Figure 2b shows that, before plasma treatment, the Bragg peaks of the praseodymia films have smaller *L* values than the Si(111) Bragg peak. Thus the praseodymia layer distance *c* is larger than the Si(111) layer distance $c_{\mathrm{Si}}$. In addition, the expected layer distances for the common praseodymia phases Pr${}_{2}$O${}_{3}$, Pr${}_{6}$O${}_{11}$ and PrO${}_{2}$ are denoted in Figure 2b. Here, we present ranges of possible Bragg peak positions for the cases ranging from pseudomorphic elastically distorted films to completely relaxed films having bulk value layer distances. Furthermore, we performed a detailed analysis of the diffraction data based on the kinematic approximation (black lines in Figure 2b) which will be discussed below.

The positions of the praseodymia Bragg peaks are larger than expected for Pr${}_{2}$O${}_{3}$ and shifted to the values expected for higher oxygen content of the praseodymia films. Thus the praseodymia films are oxidized under ambient conditions as concluded before from the SR-XRR data. This effect is well known for praseodymia powder where it is reported that Pr${}_{6}$O${}_{11}$ is the stable phase under ambient conditions [21,22].

Obviously, with increasing film thickness, the position of the praseodymia Bragg peaks shift further to higher values pointing to a decreasing layer distance. This effect can be explained by an increasing oxidation state of the praseodymia since the average size of cations decreases for higher oxidation states and the bulk lattice constant consequently shrinks (*cf.* also the noted positions of the different praseodymia phases in Figure 2b).

After plasma oxidation the positions of the Bragg peak are shifted to higher values for all investigated praseodymia films. Thus, all films have successfully been oxidized. The oxidation state of the thinnest 6 nm film, however, is far from being PrO${}_{2}$. Instead it seems, that the film finally received Pr${}_{6}$O${}_{11}$ stoichiometry although our XPS studies show the complete oxidation of the surface near region. Therefore, it seems that the interface stabilizes smaller degrees of oxidation.

On the other hand, the thicker 9 nm and 18 nm films clearly show Bragg peaks which can be attributed to PrO${}_{2}$. A detailed kinematic analysis of the diffraction data showed that the layer distance is *c* = 307 (±1) pm for both thicker films. This value is smaller than the layer distance *c* = 310 pm expected from bulk data for relaxed PrO${}_{2}$ films [21,22] and can also not be explained by elastic distortion of the film due to lattice matching at the interface. In the latter case, one would expect a layer distance of *c* = 309 pm. Thus the film seems to be “over oxidized”. However, the obtained layer distance agree well with previously published data for PrO${}_{2}$ films which have been oxidized by oxygen plasma [11].

Furthermore, it has to be emphasized that all diffraction scans except the plasma oxidized 18 nm film clearly show Laue fringes. These fringes are caused by single crystalline films with well defined film thickness and low interface and surface roughness. Generally, the intensity of the Laue fringes is smaller after plasma oxidation. Thus, the oxidation process roughens the praseodymia films. This result agrees well with our SR-XRR result (see above).

In addition, the thickness of the crystalline part of the films studied here is slightly smaller than the film thickness obtained from SR-XRR. This effect can be attributed to the non-crystalline interface layer reported before from our SR-XRR results. Furthermore, the kinematic diffraction analysis shows also that there exist crystalline interlayers which are less oxidized than the oxide film on top. This result agrees well with Gevers *et al.*, who reported residual interlayers with PrO${}_{1.833}$ stoichiometry [11].

Finally, we like to emphasize that there seems to be some residual diffraction intensity close to the Bragg position of the initial Pr${}_{6}$O${}_{11}$ film for the plasma oxidized 9 nm film. Thus, this film seems not to be completely transformed to PrO${}_{2}$. This result also agrees well with the results obtained by Gevers *et al.*, who found some lateraly separated phases in plasma oxidized praseodymia films [11]. Therefore, we performed some further studies on this film.

### 3.3. Exposure Time

We studied the effect of exposure time in a second series of experiments performed on praseodymia films of 9 nm thickness. Here, we kept the flux of 40 sccm O${}_{2}$ as well as the O${}_{2}$ pressure of 26 Pa constant.

**Figure 3:**
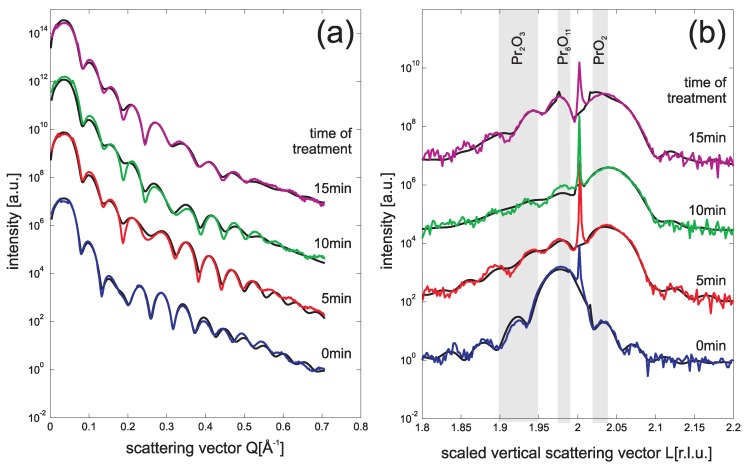
SR-XRR (**a**) and SR-XRD (**b**) data obtained from 9 nm praseodymia films after different plasma treatment times (5–15 min) keeping other parameters (26 Pa O${}_{2}$, 40 sccm flux) constant. Complete oxidation is already obtained after the first 5 min oxidation step.

In Figure 3a we present the SR-XRR data recorded after treating the praseodymia films from 5 min to 15 min. No clear changes of the SR-XRR data can be seen increasing the time of treatment. A detailed analysis fitting the data (black curves) shows that the electron density slightly increases already after the 5 min treatment. Further treatment does not cause significant improvements. Time dependent effects after the first treatment can also not be observed concerning the rms-roughness of the film as well as thickness, electron density and interface roughness of the amorphous interlayer.

Similar conclusions can also be drawn from the SR-XRD experiments on these film presented in Figure 3b. The position of the Bragg peak shifts clearly to a higher *L* value after the first treatment of 5 min. From the complete kinematic analysis we obtain that the layer distance of the praseodymia film is *c* = 306 (±1) pm pointing to a complete oxidation of the film which assumes PrO${}_{2}$ stoichiometry and structure. However, parts of the film seem still to have less oxidized stoichiometries. Furthermore, a less oxidized crystalline interface layer is detected by kinematic diffraction analysis of the SR-XRD data.

All CTRs of the plasma treated films have Laue oscillations from which we conclude that the films show homogeneous film thickness. The thickness of the crystalline parts of the film do not change with increasing treatment time.

### 3.4. Flux

In a last sequence we varied the O${}_{2}$ flux up to 80 sccm during plasma oxidation of 9 nm Pr${}_{2}$O${}_{3}$ films for 15 min in 26 Pa O${}_{2}$.

**Figure 4:**
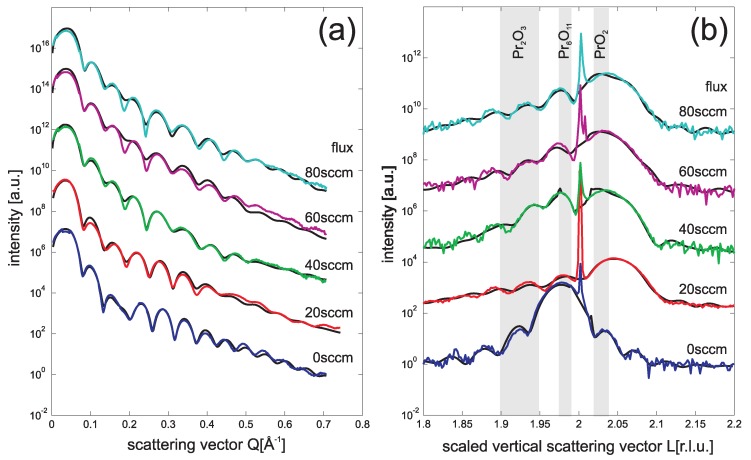
SR-XRR (**a**) and SR-XRD (**b**) data obtained from 9 nm praseodymia films using different O${}_{2}$ fluxes during plasma treatment (20–80 sccm) keeping other parameters (26 Pa O${}_{2}$, 15 min treatment time) constant. The lowest flux of 20 sccm O${}_{2}$ is alreday sufficient to completely oxidize the praseodymia film.

SR-XRR results are presented in Figure 4a. Again some small changes concerning the Kiessig fringes can be seen. A detailed analysis of the data showed that the electron density of the praseodymia continuously increases with increasing flux while the rms-roughness decreases. On the other hand increasing the flux during plasma treatment does not have any effect on the interlayer. Its thickness, roughness and electron density is independent of the flux.

Figure 4b shows the SR-XRD data for varying the flux during plasma oxidation. Even for the smallest O${}_{2}$ flux of 20 sccm, the praseodymia Bragg peak shifts to larger *L* values. From the positions of the Bragg peaks, we conclude the layer distance of *c* = 307 (±1) pm associated with the formation of PrO${}_{2}$. Finally, the clearly seen Laue fringes show that all films show homogeneous thickness.

## 4. Conclusions

**Figure 5:**
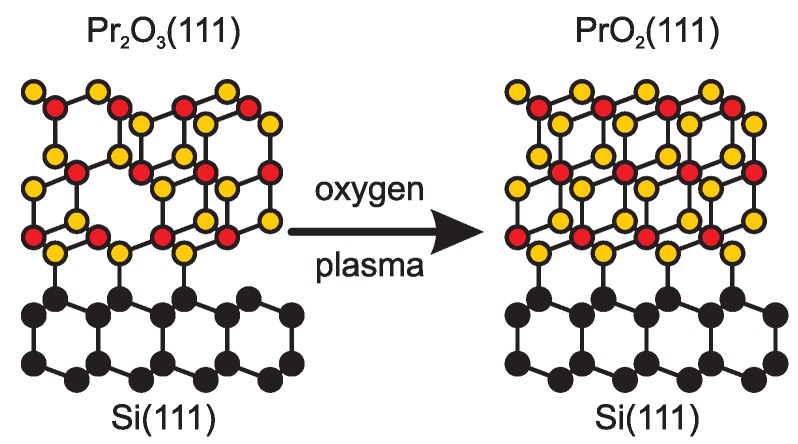
Schematic drawing of the plasma oxidation process. Randomly distributed oxygen vacancies in the Pr${}_{2}$O${}_{3}$ film are occupied by oxygen from the oxygen plasma. The drawing considers also the B orientation of the praseodymia films with respect to the Si(111) support.

In summary, we have studied the O${}_{2}$ plasma oxidation of Pr${}_{2}$O${}_{3}$ films by means of LEED and XPS as well as synchrotron radiation based SR-XRR and SR-XRD. The results demonstrate that the praseodymia films can easily be transformed from Pr${}_{2}$O${}_{3}$ to PrO${}_{2}$ after exposure to O${}_{2}$ plasma (*cf.* Figure 5). The surface near region always shows PrO${}_{2}$ structure and stoichometry independent of film thickness, exposure time and flux. On the one hand, PrO${}_{2}$ oxidation can also be verified by SR-XRR and SR-XRD if the initial film thickness exceeds 6 nm. This effect is clearly seen already for the smallest exposure times of 5 min and for the smallest O${}_{2}$ fluxes of 20 sccm. On the other hand, the bulk of 6 nm Pr${}_{2}$O${}_{3}$ films cannot completely be oxidized as demonstrated by SR-XRD. Here, we obtain a larger layer distance which is associated with the formation of Pr${}_{6}$O${}_{11}$ stoichiometry. Finally, we like to emphasize that oxidation by oxygen plasma leads to the higher oxidation states compared to oxidation by molecular O${}_{2}$.

## References

[1] Bhaskaran K., Bhat V.T. (2002). Catalytic activity of CeO_2_ and Pr_2_O_3_ for the liquid-phase benzylation of o-xylene to 3,4-dimethyldiphenylmethane. React. Kinet. Catal. Lett..

[2] Bedell S.W., Reznicek A., Fogel K., Ott J., Sadana S.K. (2006). Strain and lattice engineering for GeFET devices. Mater. Sci. Semicond. Process..

[3] Tanoto H., Yoon S.F., Loke W.K., Fitzgerald E.A., Dohrman C., Narayanan B., Doan M.T., Tung C.H. (2006). Growth of GaAs on vicinal Ge surface using low-temperature migration-enhanced epitaxy. J. Vac. Sci. Technol..

[4] Giussani A., Seifarth O., Rodenbach P., Müssig H.-J., Zaumseil P., Weisemoeller T., Deiter C., Wollschläger J., Storck P., Schroeder T. (2008). The influence of lattice oxygen on the initial growth behavior of heteroepitaxial Ge layers on single crystalline PrO_2_(111)/Si(111) support systems. J. Appl. Phys..

[5] Seifarth O., Walczyk Ch., Lupina G., Dabrowski J., Zaumseil P., Weidner G., Müssig H.-J., Schroeder T. (2009). Dielectric properties of single crystalline PrO_2_(111)/Si(111) heterostructures: Amorphous interface and electrical instabilities. J. Appl. Phys..

[6] Schroeder T., Lee T.-L., Libralesso L., Joumard I., Zegenhagen J. (2005). Structure and strain relaxation mechanism of ultrathin epitaxial Pr_2_O_3_ films on Si(111). J. Appl. Phys..

[7] Weisemoeller T., Deiter C., Bertram F., Gevers S., Giussani A., Zaumseil P., Schroeder T., Wollschläger J. (2008). Epitaxy of single crystalline PrO_2_ films on Si(111). Appl. Phys. Lett..

[8] Weisemoeller T., Bertram F., Gevers S., Greuling A., Deiter C., Tobergte H., Neumann M., Wollschläger J., Giussani A., Schroeder T. (2009). Post deposition annealing induced transition from hexagonal Pr_2_O_3_ to cubic PrO_2_ films on Si(111). J. Appl. Phys..

[9] Gevers S., Weisemoeller T., Zimmermann B., Bertram F., Deiter C., Wollschläger J. (2009). Structural Phase Transition of Ultra Thin PrO_2_ Films on Si(111). J. Phys. Condens. Matter.

[10] Schaefer A., Gevers S., Zielasek V., Schroeder T., Falta J., Wollschläger J., Bäumer M. (2011). Photoemission study of praseodymia in its highest oxidation state: The necessity of *in situ* plasma treatment. J. Chem. Phys..

[11] Gevers S., Weisemoeller T., Schaefer A., Zielasek V., Bäumer M., Wollschläger J. (2011). Structure of oxygen-plasma-treated ultrathin praseodymia films on Si(111). Phys. Rev. B.

[12] Schroeder T., Zaumseil P., Weidner G., Wenger Ch., Dabrowski J., Müssig H.-J., Storck P. (2006). On the epitaxy of twin-free cubic (111) praseodymium sesquioxide films on Si(111). J. Appl. Phys..

[13] Liu J.P., Zaumseil P., Bugiel E., Osten H. (2001). Epitaxial growth of Pr_2_O_3_ on Si(111) and the observation of hexagonal to cubic phase transition during postgrowth N_2_ annealing. Appl. Phys. Lett..

[14] Ogasawara H., Kotrani A., Potze R., Sawatzky G.A., Thole B.T. (1991). Praseodymium 3d- and 4d- core photoemission spectra of Pr_2_O_3_. Phys. Rev. B.

[15] Kotani A., Ogasawara H. (1992). Theory of core level spectroscopy of rare earth oxides. J. Electron Spectrosc. Relat. Phenom..

[16] Bianconi A., Kotani A., Okada K., Giorgi A., Marcelli A., Miyahara T. (1988). Many-body effects in praesodymium core-level spectroscopies of PrO_2_. Phys. Rev. B.

[17] Hu Z., Kaindl G., Ogasawara H., Kotani A., Felner I. (2000). Ln-4f/ligand-2p covalence in BaLnO_3_ and Cs_3_LnF_7_ (Ln = Ce, Pr, Tb). Chem. Phys. Lett..

[18] Parratt L.G. (1954). Surface studies of solids by total reflection of X-rays. Phys. Rev..

[19] Nevot L., Croce P. (1980). Characterisation of surfaces by grazing X-ray reflection. Application to the study of polising some silicate glasses. Rev. Phys. Appl..

[20] Weisemoeller T., Bertram F., Gevers S., Deiter C., Greuling A., Wollschläger J. (2009). Effect of amorphous interface layers on crystalline thin-film X-ray diffraction. Phys. Rev. B.

[21] Eyring L., Gschneidner K.A., Eyring L. (1979). The binary rare earth oxides. Handbook on the Physics and Chemistry of Rare Earths.

[22] Haire R.G., Eyring L., Gschneidner K.A., Eyring L., Choppin G.R., Lander G.H. (1994). Comparison of the binary oxides. Handbook on the Physics and Chemistry of Rare Earths.

